# Nitrogen metabolism in haloarchaea

**DOI:** 10.1186/1746-1448-4-9

**Published:** 2008-07-01

**Authors:** María José Bonete, Rosa María Martínez-Espinosa, Carmen Pire, Basilio Zafrilla, David J Richardson

**Affiliations:** 1 División de Bioquímica y Biología Molecular, Facultad de Ciencias, Universidad de Alicante, Ap. 99, E-03080, Alicante, Spain; 2 School of Biological Sciences, Faculty of Science, University of East Anglia, Norwich, NR4 7TJ, UK

## Abstract

The nitrogen cycle (N-cycle), principally supported by prokaryotes, involves different redox reactions mainly focused on assimilatory purposes or respiratory processes for energy conservation. As the N-cycle has important environmental implications, this biogeochemical cycle has become a major research topic during the last few years. However, although N-cycle metabolic pathways have been studied extensively in *Bacteria *or *Eukarya*, relatively little is known in the *Archaea*. Halophilic Archaea are the predominant microorganisms in hot and hypersaline environments such as salted lakes, hot springs or salted ponds. Consequently, the denitrifying haloarchaea that sustain the nitrogen cycle under these conditions have emerged as an important target for research aimed at understanding microbial life in these extreme environments.

The haloarchaeon *Haloferax mediterranei *was isolated 20 years ago from Santa Pola salted ponds (Alicante, Spain). It was described as a denitrifier and it is also able to grow using NO_3_^-^, NO_2_^- ^or NH_4_^+ ^as inorganic nitrogen sources. This review summarizes the advances that have been made in understanding the N-cycle in halophilic archaea using *Hfx mediterranei *as a haloarchaeal model. The results obtained show that this microorganism could be very attractive for bioremediation applications in those areas where high salt, nitrate and nitrite concentrations are found in ground waters and soils.

## Background

**Figure 1:**
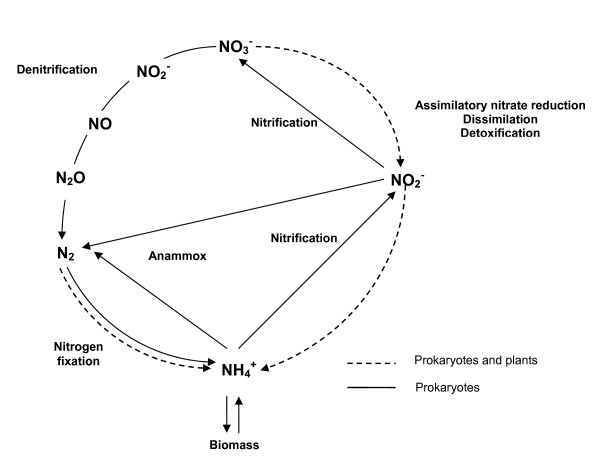
N-cycle scheme. NO_3_^- ^is used as nitrogen source for growth under aerobic conditions using an *assimilatory NO*_3_^- ^*reductase*, while it acts as an electron acceptor to eliminate excess of reductant power through *dissimilatory NO*_3_^- ^*reduction*. *Dissimilatory NO*_3_^- ^*reduction*, *NO*_3_^- ^*respiration *or *denitrification *are often used equivalently in the literature. However, *dissimilatory *pathway makes reference to non-assimilatory reactions that are not directly coupled to generation of proton-motive force. In some Enterobacteriaceae, NO_2_^- ^is reduced to NH_4_^+ ^which is then excreted; this process is known as *NO*_3_^-^/*NO*_2_^- ^*ammonification*. Specialised organisms are able to oxidize either NH_4_^+ ^or NO_2_^- ^by using a pathway called *nitrification*, while other organisms such as some planctomycetes oxidize NH_4_^+ ^and utilize NO_2_^- ^as respiratory electron acceptor in a pathway named *anammox*. Finally, (*di)nitrogen fixation *allows several bacteria and archaea to reduce N_2 _to NH_4_^+ ^to provide N-requirements.

Nitrogen (N) is a major element in all organisms. It accounts for approximately 6% of their dry mass on average and thus in nature its assimilation is a key process of the N-cycle carried out by higher plants [1], algae [2], yeast [3], and bacteria [4]. In the environment, N can be found in different redox states from +5 (as nitrate) to -3 (as ammonia), but in biological compounds it is almost exclusively present in the most reduced form as a component of the two pre-eminent biological macromolecules: proteins and nucleic acids [5]. The reactions of the global biogeochemical N-cycle makes possible the interconvertions of nitrogen compounds and it includes both reductive and oxidative processes, in which prokaryotes play a predominant role (Fig. 1).

The assimilatory pathways of the N-cycle (*N*_2 _*fixation *and *nitrate assimilation*) generate ammonium that is then incorporated into the carbon skeletons to produce amino acids. However, whereas N_2 _fixation is carried out by free-living or symbiotic diazotrophic prokaryotes, assimilatory nitrate reduction is a property of many species of bacteria, fungi, algae, higher plants and Archaea. *Nitrification *and *denitrification *are redox processes involving nitrogen compounds to obtain metabolic energy. Nitrification consists of the oxidative conversion of ammonia to nitrite via hydroxylamine, and further oxidation of nitrite to nitrate [6]. On the other hand denitrification is a respiratory process (under anaerobic conditions) whereby nitrate is reduced to nitrite, NO, N_2_O and N_2_. *Ammonification *is the dissimilatory reduction of nitrate to ammonia that does not serve the purpose of nitrogen autotrophy [7]. *Anaerobic ammonium oxidation *(Anammox) is a reaction that produces N_2 _by reducing nitrite and oxidizing ammonium. This process, recently described, seems to be of ecological importance in marine environments [8].

Some of the compounds produced thanks to N-cycle reactions could affect life in different ways. Related to this, nitrous oxide (N_2_O) and nitric oxide (NO) have impact on the atmosphere: i) N_2_O is a more potent greenhouse gas than CO_2 _contributing to global warming, ii) in the stratosphere, N_2_O and NO destroy the ozone layer that protects organisms against UV light, iii) NO can be chemically oxidized to NO_2_, which is further hydrated to HNO_2 _and HNO_3 _(components of the acid rain). Besides, it is very important to note that when fertilizers are used at high concentrations or are not used by plants or microorganisms, products such as NO_3_^- ^or NO_2_^- ^can enter the aquifers [9,10]. The consumption of drinking water containing high NO_3_^- ^and NO_2_^- ^concentrations causes human health concerns because NO_2_^- ^interacts with haemoglobin. This results in the inhibition of the oxygen transport through the human body which is known as metahemoglobinemia. It has also been demonstrated that different kinds of gastric cancer are associated with the consumption of water with high nitrate and nitrite concentrations [11].

Although the N-cycle is well characterised in bacteria from physiological, biochemical and genetic points of view, there are few studies centred on this metabolic pathway in halophilic archaea. These studies, carried out with several *Haloferax, Haloarcula *and *Halobacterium *species, are mainly focused on denitrification [12-17]. Related to this, *Hfx mediterranei *is the only halophilic archaeon from which assimilatory pathway has been analysed in detail [18-22]. Recent studies on the respiratory nitrate pathway have revealed that respiratory nitrate reductases from *Hfx mediterranei *or *Har marismortui *are facing on the positive face of the membrane, instead of having the active site facing the cytoplasm as it has been reported for most of the bacterial nitrate reductases [16,23]. This review covers the current knowledge on N-cycle in *Hfx mediterranei *with the objective to shed light on the haloarchaeal nitrogen metabolism and its implications on several environmental issues.

### Assimilatory nitrate reduction in *Hfx mediterranei*

Nitrate assimilation is one of the main processes of the N-cycle, and it allows the use of NO_3_^- ^as N source for growth. *In silico *studies have revealed that genes encoding the proteins involved in nitrate assimilation have been found in the genomes of the two major *Archaea *subgroups: crenarchaeota and euryarchaeota [24]. However, physiological and biochemical characterisation of NO_3_^- ^assimilation have been performed only in *Hfx mediterranei *at the time of writing this review.

In the assimilatory nitrate reduction, first NO_3_^- ^is incorporated into the cells by high-affinity transporters and further reduced to NH_4_^+^, via NO_2_^-^, by two sequential reduction reactions catalysed by assimilatory nitrate reductase (Nas; EC 1.6.6.2) and assimilatory nitrite reductase (Nir; EC 1.7.7.1). The NH_4_^+ ^produced is incorporated into carbon skeletons by the glutamine synthetase/glutamate synthase pathway (GS-GOGAT; EC 6.3.1.2, EC 1.4.7.1, respectively) or via glutamate dehydrogenase (GDH; EC 1.4.1.2). The GS/GOGAT pathway is particularly important because it allows ammonia assimilation into L-Glu at low intracellular ammonia concentrations and it seems that it efficiently substitutes the other glutamate biosynthetic reaction (GDH) in these conditions. Nevertheless, in some bacteria it has been found that GDH is not significantly affected by the type or the concentration of the nitrogen source supplied [25].

#### NO_3_^- ^uptake

In bacteria, the genes coding for the regulatory and structural proteins required for NO_3_^- ^uptake and reduction are, in most cases, clustered [26] and frequently NO_3_^- ^is transported into the cells by an active system. Two types of nitrate transporters are involved in prokaryotic assimilatory nitrate reduction: ATP-dependent ABC transporters (composed of an integral membrane subunit, a cytoplasmic ATP-binding component and a periplasmic substrate-binding protein) and the monomeric NarK-type transporters belonging to the major facilitator superfamily (MFS-type permease), which depend on proton-motive force. ABC transporters form a widespread protein family present in Archaea, Bacteria and Eukarya. In the haloarchaeal *Hfx volcanii*, three genomic regions containing genes coding for ABC transporters subunits involved in nitrate respiration were characterised [27], but there is not any evidence of its participation in the assimilatory process. These genes have been characterised from genomic libraries contructed using several nitrate respiration-deficient *Hfx volcanii *mutants. Most archaea with putative ABC nitrate transporters seem to contain respiratory nitrate reductases rather than assimilatory nitrate reductases.

Within the bacterial NarK-like transporters there are two subgroups: NarK1 (proton: nitrate symporter that allows initiation of nitrate respiration) and NarK2 (nitrate: nitrite antiporter required for maintenance of a steady-state rate) [28]. Some of these proteins are involved in NO_3_^-^/NO_2_^- ^exchange rather than simply in the uptake of one of these anions, but the mechanism of the transport has not been determined for any of these bacterial MFS importers. It has been suggested that bacterial nitrate assimilation usually requires ATP-dependent ABC nitrate transporters whereas nitrate respiration is associated with proton-motive-force driven NarK transporters [28].

Database comparisons of the genes involved in the assimilation of nitrate in *Hfx mediterranei *revealed that *nasB *gene (Q703N4) encodes a NO_3_^- ^transporter with a molecular mass around 46.1 kDa. This transporter is a membrane protein with 12 potential α helices and is most closely related with the NarK1 type of transporters. The best fits of *nasB *gene were with bacterial homologues, such as *Thermus thermophilus*, *Paracoccus halodenitrificans *and *Pseudomonas aeruginosa *[20]. *nasB *from *Hfx mediterranei *is the first NarK transporter reported to date in Archaea, which suggests that the NarK group could be involved in the assimilatory process and it is not exclusive to *Bacteria *and *Eukarya *as it had originally been suggested.

#### Nitrate and nitrite assimilation

**Figure 2:**
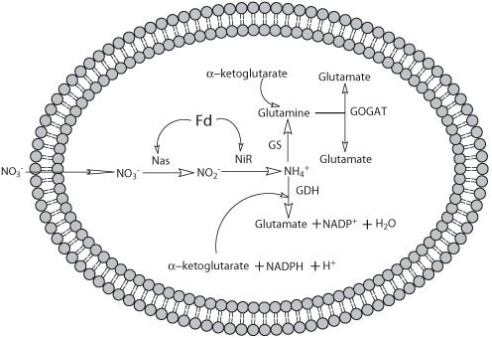
Assimilatory nitrate reduction in *Hfx mediterranei*. All the enzymes presented in the figure have been purified and characterised except glutamate synthase. Nas: assimilatory nitrate reductase; NiR: assimilatory nitrite reductase; Fd: Ferredoxin; GDH: glutamate dehydrogenase; GS: glutamine synthetase; GOGAT: glutamate synthase.

When nitrate is imported into *Hfx mediterranei *cells, it is reduced to nitrite by the ferredoxin dependent assimilatory nitrate reductase (Nas). In general, Nas are cytoplasmic enzymes that catalyse the two-electron reduction of NO_3_^- ^to NO_2_^-^. They are repressed by ammonium and use either NADH or ferredoxin as physiological electron donors, although some use flavodoxin instead of ferredoxin. Fd-Nas are usually monomeric enzymes while NADH-dependent Nas have been described as heterodimeric proteins [29]. Both of them are structurally and functionally different from the dissimilatory periplasmic nitrate reductases (Nap; EC 1.7.99.4) and the respiratory membrane-bound nitrate reductases (Nar; EC 1.7.99.4) present in many prokaryotes. On the basis of the gene sequence and the UV-Vis spectra, *Hfx mediterranei *ferredoxin-dependent Nas (Q703N5) contains a Mo-bis-molybdopterin guanine dinucleotide cofactor (Mo-bis-MGD) and one [4Fe-4S] cluster [19,20]. In this case, the electrons probably flow from the [2Fe-2S] cluster-containing ferredoxin (which is a negative redox potential electron donor) to the [4Fe-4S] cluster and from this centre to the Mo-cofactor for the reduction of NO_3_^- ^(Fig. 2). *Hfx mediterranei *Nas was first described as a dimeric enzyme [19]. Nevertheless, recent studies have revealed that this enzyme is a monomeric protein with a molecular mass around 75 kDa and it is most closely related with monomeric bacterial ferredoxin-dependent Nas proteins [20]. The highest similarity scores were to the Nas proteins of *Pseudomonas aeruginosa*, *Xanthomonas campestris *and *Synechococcus elongates *[20]. The comparison with the products of the putative assimilatory nitrate reductase genes from other archaea showed that there was only a low overall similarity between these and assimilatory nitrate reductase from *Hfx mediterranei*, with conserved residues predominantly being associated with the cofactor binding sites. Nas kinetic parameters have been obtained that suggest that the *K*_*m *_for nitrate is around 0.95 mM and the enzyme has maximum activity at 80°C in 3.1 M NaCl, but 60°C in 1.3 M NaCl. Nas can receive electron from methylviologen and benzylviologen but not NAD(P)H. Nas activity is induced by nitrate and repressed by ammonium, as described for bacterial Nas [20,22]. Up to now, this is the only Nas purified and characterised from a biochemical and genetical point of view from haloarchaea.

Nitrite produced by Nas is reduced to ammonium by ferredoxin dependent assimilatory nitrite reductase (Q703N2) that catalyses the six-electron reduction of NO_2_^-^. As it has been described above for Nas, assimilatory nitrite reductases are classified into two groups according to the electron donor specificity: Fd-dependent Nir (described from eukaryotic and prokaryotic photosynthetic organisms) and NAD(P)H-dependent Nir (present in fungi and most heterotrophic bacteria). Fd-Nirs are cytoplasmic monomeric proteins with molecular mass around 55 kDa. They contain a siroheme and a [4Fe-4S] cluster as redox centres. The electrons flow from ferredoxin to Nir in a similar way to that described for Nas (electrons from the [2Fe-2S] cluster-containing ferredoxin are transferred to the [4Fe-4S] cluster and from this centre to the siroheme for the reduction of nitrite). On the other hand, NADH-Nirs consist of two subunits, and contain non covalently bound FAD, a [4Fe-4S] cluster, and siroheme as prosthetic groups. The assimilatory nitrite reductase purified from *Hfx mediterranei*, the first one purified from this kind of microorganisms, is a 66 kDa monomer that shows a strong sequence similarity with known ferredoxin-dependent nitrite reductases, such as those of *Synechocystis sp*., *Plectonema boryanum *and *Synechococcus elongatus*. Some of its biochemical properties have been analysed in detail showing that it has a *K*_*m *_for nitrite of 8.6 mM, and maximal activity at 60°C in presence of 3.3 M NaCl. Like most of the bacterial assimilatory nitrite reductases described so far, the *Hfx mediterranei *Nir contains siroheme and Fe-S centres [18,20].

The *Hfx mediterranei *ferredoxin involved in the electron transfer to Nas or Nir is a small protein (Mr ≈ 21 kDa on SDS-PAGE) [30,31] that shows UV-Vis, NMR and EPR spectra similar to those described from plant and bacteria ferredoxins containing one [2Fe-2S] cluster. The electron paramagnetic resonance spectrum of the reduced form of the protein displayed a rhombic signal, with gx = 1.91, gy = 1.98, gz = 2.07. UV-visible spectropotentiometric analysis determined a midpoint redox potential for the [2Fe-2S]^2+/1+ ^transition of around -285 mV vs. SHE that was independent of salt concentration. Significantly, this ferredoxin was shown to be a highly thermostable protein. It was stable up to 60°C in a low-salt (0.2 M) medium and this increased to 80°C in a high-salt (4 M) medium. Ferredoxins from other halophilic archaea such as *Hbt halobium *[32], *Har marismortui *[33], and *Har japonica *[34] have been purified and characterised and in all cases they contain a [2Fe-2S] cluster. However, although all of them belong to the plant type differences between these two groups have been emphasised [32].

#### Ammonium transport and assimilation

When ammonium is the nitrogen source for growth, the first step is its across biological membranes facilitated by the ammonium transporter (Amt). Amt transporters are integral membrane proteins that allow the ammonium and methylammonium uptake. This kind of transporter is well characterised in *Bacteria *and *Eukarya *domains, however it still remains quite unknown in *Archaea*, in fact only the Amt from *Archaeoglobus fulgidus *[35] has been studied to date from the *Archaea *domain. Recent structural information on two prokaryotic Amt proteins has significantly contributed to our understanding of these transporters [36]. The mechanisms involved in the ammonium uptake in *Hfx mediterranei *are unknown at the time of writing this paper. However, the processes related to the assimilation of ammonium produced by Nir in this archaea have been recently described in our group [22].

Glutamine synthetase-glutamate synthase (GS-GOGAT) and glutamate dehydrogenase (GDH) are the main pathways for ammonium assimilation in several microorganisms including *Hfx mediterranei*. The first pathway requires ATP but has high affinity for ammonium, whereas GDH does not consume ATP but is less effective in cells growing in N-limited conditions. These enzymes are present in all three domains of life.

Glutamine synthetase (GS), the first enzyme of the GS/GOGAT pathway, has been extensively studied at both biochemical and molecular levels in prokaryotes, mammals and some methanogenic and hyperthermophilic archaea [37,38]. This enzyme plays a dual role by providing glutamine for biosynthesis and by assimilating ammonium in collaboration with glutamate synthase. In summary, three types of GS have been described based on the molecular size and the number of subunits. GSI is a dodecameric protein with subunit ranging between 44 and 60 kDa, and is usually found among several groups of bacteria and archaea [39,40]. GSII is common in eukaryotes and a few soil-dwelling bacteria as an octameric enzyme composed of subunits ranging between 35 and 50 kDa [41]. Finally, GSIII has been characterised from cyanobacteria and two anaerobic bacteria as a hexameric enzyme composed of a 75 kDa subunits [42].

Few analyses have been carried out with GS from members of *Archaea*, and just two are from GS from haloarchaea [21,43]; these enzymes, purified from *Hfx mediterranei *and *Hbt salinarium *are octamers belonging to the GS type II [21,43]. However, a few GSs described from methanogenic or hyperthermophilic archaea are dodecamers of about 600 kDa. So, GS from haloarchaea exhibits typical properties of GS from eukaryotes and soil bacteria species. This fact supports the hypothesis that some members of Archaea are quite like eukaryotic organisms because both of them share similar properties at physiological and metabolic levels [44]. The results obtained from *Hfx mediterranei *suggest that GS from this haloarchaea could allow the assimilation of the ammonium produced by assimilatory nitrite reductase [21,22], while GDH would allow the assimilation of ammonium when this nitrogen source is present in the culture media at high concentrations. The biochemical characterisation of *Hfx mediterranei *GS shows an optimum pH value for activity around 8. Either in the presence of NaCl or KCl, the maximum stability was found at the highest salt concentration described by us (3.5 M and 2.5 M, respectively). The *K*_*m *_value for ADP could not be calculated for the transferase activity, as all concentrations analysed produced similar results (*K*_*m *_around 3.10 ± 0.5 mM). The apparent *K*_*m *_for NH_2_OH, Gln, ATP and Glu were 10.5 ± 3.5, 25 ± 1.8, 0.30 ± 0.08 and 4.9 ± 1.5 mM, respectively. These values are similar to those described in *Hbt salinarium *GS and are in the range described for GS from different cyanobacteria [45] and Archaea [46]. GS activity decreased substantially in the presence of MSX (an irreversible inhibitor of bacterial GS) as it was described from other prokaryotic GS [22]. Studies about the effectiveness of different metal ions on the activity assay revealed that Mn^2+ ^was the most effective ion for transferase activity as it was expected due to its essential role as a cofactor.

Glutamate synthase, the second enzyme of the GS/GOGAT cycle, is an NADPH dependent enzyme in most of bacteria and it has two dissimilar enzyme subunits α and β that forms the αβ active protomer which contains one FAD, one FMN, and three different [Fe-S] centres: one [3Fe-4S] cluster and two [4Fe-4S] centres [47]. The plant type GOGAT is dependent on reduced ferredoxin as physiological electron donor and it is formed by a single polypeptide chain similar in sequence and cofactor content (one FMN, and one [3Fe-4S] cluster) to the α subunit of the bacterial enzyme.

Whether *Archaea *contain a bacterial type, a plant type or a fourth class still needs to be verified. *Methanococcus jannaschii *genome sequencing first revealed the presence of one ORF encoding a 490 residues polypeptide that appears to be formed by an N-terminal domain containing the Cys-signature typical of two bacterial ferredoxins [4Fe-4S] clusters followed by a polypeptide mapping on the synthase domain of NADPH-GltS α subunit [47]. A similar ORF has been found in *A fulgidus *and appears to be conserved in other *Archaea *and in *Thermatogales *as the result of lateral gene transfer. It has also been proposed that a fifth type of glutamate synthase may exist. An ORF encoding a 50 kDa protein of *Pyrococcus *sp. KOD1 with significant sequence similarity to NADPH-GltS β subunit has been found and it was assigned the gene name of *glt*A [47]. The analysis of the halophilic archaeal genomes sequenced suggested that in *Har marismortui, Natronomonas pharaonis *and *Haloquadratum walsbyi *there are open reading frames of about 1500 residues similar in sequence to the plant type ferredoxin dependent glutamate synthase and to the bacterial NADPH glutamate synthase α subunit, but there is not evidence of the presence of an open reading frame corresponding to a polypeptide similar to the bacterial NADPH glutamate synthase β subunit. In *Hfx mediterranei*, we have detected activity of glutamate synthase with methylviologen as reducing agent in extracts from cells grown with ammonium starvation (data not published). Analysis of the gene sequence revealed a high degree of homology with ORFs of sequenced genomes of Fd-dependent glutamate synthases. The classification of archaeal glutamate synthase and particularly of haloarchaeal glutamate synthase stills needs to be verified by biochemical characterization.

As it has been cited before, *Hfx mediterranei *is able to assimilate ammonium via glutamate dehydrogenase (GDH) [48]. This is an important enzyme because it catalyses the interconvertion between 2-oxoglutarate and L-glutamate reversibly and plays a key role since it provides a link between carbon and nitrogen metabolism. Several studies have demonstrated that under nitrogen-sufficient conditions, GDH mainly catalyses glutamate production from 2-oxoglutarate and ammonium. However, under nitrogen-starvation conditions, GS produces glutamine from glutamate and ammonium, and glutamine is then converted to glutamate by GOGAT. The GS-GOGAT pathway is the major route for utilization of ammonium when ammonium is deficient [49] because GS has a much lower *K*_*m *_for ammonium than does GDH. However, in some bacteria it has been found that GDH is active under low ammonium conditions [25].

GDHs are classified into three groups according to their coenzyme specificity: NAD or NADP-specific and NAD(P)-non specific dependent GDH. *Hfx mediterranei *has at least two different GDHs: NADP-GDH and NAD-GDH [48,50].

### Assimilatory nitrate pathway regulation in *Hfx mediterranei*

In *Hfx mediterranei *a 6,720 bp genomic fragment was sequenced containing homologues of the nitrate assimilation genes *nasABC *and *D*, which code for assimilatory nitrate reductase (*nasA*, Q703N5), a nitrate transporter belonging to NarK group (*nasB*, Q703N4), cited above, a molybdopterin guanine dinucleotide biosynthesis protein (*nasC*, Q703N3) and an assimilatory nitrite reductase (*nasD*, Q703N2). RT-PCR studies demonstrated [21] that *nasABC *are cotranscribed but *nasD *is transcribed as a monocistronic messenger which is a novel organization in comparison with bacterial *nas *operons where, in general, bacterial nitrite reductase gene is found within the nitrate reductase gene operon [51]. The location of *Hfx mediterranei *nitrite reductase gene outside of *nas *operon raises interesting questions regarding the regulation of *nasABC *and *nasD *in relation to the cellular demand for nitrogen assimilation. Both promoters show a good match to the archaeal consensus TATA box, and transcription factor B responsive element. Moreover, palindromic regions were identified that may be implicated in regulation. Taken together, both promoters could be controlled by common and different modulators.

In ammonium medium, transcription of *nasABC *or *nasD *was not detected, but in minimal medium with nitrate both were transcribed, indicating that these genes were coding for a nitrate assimilation system. Moreover, *nasABC *mRNA was detected in minimal medium supplemented with nitrite, suggesting that the absence of ammonium was the effector of expression. The results obtained from physiological experiments were in agreement with those obtained from the analysis of the expression of *nasA *and *nasD *genes, i.e, ammonium repressed the expression of the assimilatory genes. However, in the presence of nitrate or nitrite as nitrogen sources, *nasA *and *nasD *expression levels increased under different culture conditions. This mechanism is a general control, which responds to the availability of the preferred nitrogen source: ammonium.

In addition to the short-term inhibitory effect of ammonium on nitrate uptake, ammonium can exert a negative effect on nitrate assimilation by affecting the activity levels of the enzymes involved in the assimilatory pathway. This ammonium effect, which was described many years before in bacteria and cyanobacteria [52], requires a more prolonged time scale to be expressed. Results obtained from *Hfx mediterranei *suggest that ammonium also causes the same long-term effect on both assimilatory activities, Nas and NiR. In cyanobacteria, the simultaneous presence in the medium of nitrate and ammonium is equivalent to that of ammonium alone [52]. So, the regulatory pattern of the assimilatory pathway in *Hfx mediterranei *is similar to those described for halotolerant bacteria [49], bacteria [4] and cyanobacteria [52], i.e., ammonium represses *nas *gene expression and inhibits the nitrate assimilation pathway, wheras nitrate is an inducer of gene expression. Accordingly, it has been observed that in the presence of ammonium nitrate or ammonium nitrite, ammonium is preferentially consumed by *Hfx mediterranei *cells.

New strategies should be addressed in the next few years in order to elucidate the mechanisms and the proteins involved in the regulation of the assimilatory metabolic pathway in *Hfx mediterranei*.

### Denitrification

Denitrification, the biological production of NO, N_2_O and N_2 _gases from NO_3_^- ^under anoxic conditions, is a key process that contributes to the nitrogen cycle of the Earth [53]. In this pathway, nitrate may be reduced to N_2 _thanks to the reactions catalysed by respiratory nitrate reductase, respiratory nitrite reductase, nitric oxide reductase and nitrous oxide reductase. Each of these enzymes is coupled to energy-conserving electron-transport pathways [54]. Denitrifying microorganisms are distributed among the three domains of the life: Archaea, Bacteria and Eukarya and are able to respire oxygen, indicating a close evolutionary relationship between the two processes, where denitrification has been considered so far the ancestor of aerobic respiration [55,56]. The ability to use nitrate as a terminal electron acceptor in energy metabolism is found in several halophilic and hyperthermophilic archaea: many of them can perform the entire denitrification process. However this process has only been partially described from biochemical or genetically point of views from some haloarchaea species (*Halobacterium *sp., *Hfx mediterranei*, *Hfx denitrificans *and *Har marismortui)*.

#### Nitrate reduction

Membrane-bound nitrate reductase (Nar) is the enzyme involved in anaerobic nitrate respiration and denitrification, being negatively regulated by O_2 _and unaffected by ammonium. These enzymes are composed of a catalytic subunit that binds a complex organic cofactor [the bis-molybdopterin guanine dinucleotide (bis-MGD) cofactor] (NarG) and an electron-transfer subunit with four iron-sulphur centres (NarH). In most of bacteria, this complex bound to a membrane dihaem *b *quinol-oxidizing component (NarI). Nar enzymes have been purified from several denitrifying haloarchaea belonging to *Haloferax *and *Haloarcula *genera. In some cases such us *Hfx denitrificans*, *Har marismortui *and *Hfx mediterranei*, the enzyme has been purified as a heterodimeric protein composed of two subunits with molecular masses around 116, 117, or 112 kDa (Nar G) and 60, 47 or 61 kDa (NarH), respectively [15]. In other species such as *Hfx denitrificans *[14] or *Hfx volcanii *[13], Nar has been described as a heterotrimeric (100, 61 and 31 kDa) protein. In *Hfx mediterranei *two different nitrate reductases involved in non-assimilatory processes have been reported: Nar (characterised in our group) and a dissimilatory nitrate reductase studied by Alvarez-Ossorio et al [12]. The last one seems to be a protein with higher molecular mass and most of its enzymatic properties are different to those described from the enzyme characterised in our laboratory [15], so it is possible to think that the enzyme purified by Alvarez, described as a dissimilatory protein, allows the dissipation of reducing power for redox balancing. This possible role should be addressed by physiological analysis.

The N-terminal region of *Har marismortui *and *Hfx mediterranei *NarG includes a typical twin-arginine signal peptide for protein translocation across the membrane by TAT export pathway (twin arginine-dependent translocase) [23], and both enzymes have activity *in situ *with both, membrane permeable benzylviologen and membrane-impermeable methylviologen, suggesting that the catalytic site is located on the outside of the membrane.

**Figure 3:**
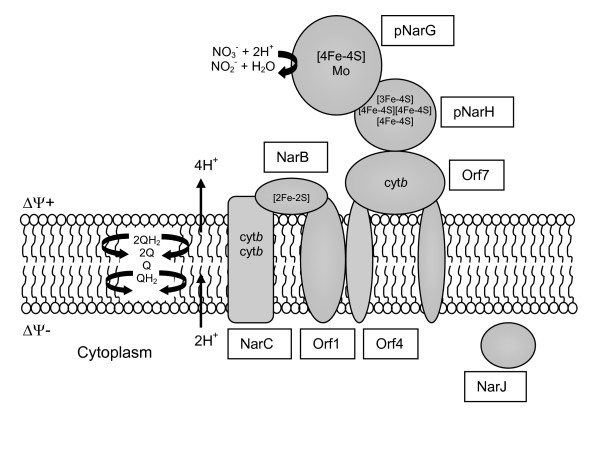
Orientation of the NarGH complex in *Hfx mediterranei *membranes. Informatic analysis of the nar operon as well as NarGH activity assays carried out with whole cells have revealed that archaeal Nars are a new type of enzyme with the active site facing the positive side of the membrane (pNarG).

Therefore, based on subunit composition, subcellular location of the active site and *nar *gene organization, it can be concluded that archaeal Nars are a new type of enzyme with the active site facing the outside and anchored to the membrane by a cytochrome *b *(as it has been proposed for *Hfx mediterranei *system) or stabilized by the lipid environment in the membrane as described for the *P. aerophilum *Nar [57]. This system could be an ancient respiratory nitrate reductase, although the nitrite formed could also be assimilated. The outside location of the catalytic site of NarG in the halophilic archaeal Nar has important bioenergetic implications because to be energy-conserving require the coupling of this process to a proton-motive complex [23], instead of the typical redox-loop mechanisms, the NarI subunit described in bacteria. On the other hand, it seems that an active nitrate-uptake system would not be required for respiratory nitrate reduction in archaea, thus increasing the energetic yield of the nitrate reduction process (Fig 3).

#### Nitrite reduction

Nitrite produced by Nar is reduced to NO by the respiratory nitrite reductases. This reaction also takes place under anoxic conditions and it is not as well known as nitrate respiration in haloarchaea. In bacteria two different respiratory nitrite reductases have been reported: the homotrimeric copper-containing enzyme (encoded by *nirK*) and the homodimeric cytochrome cd_1_-nitrite reductase (encoded by *nirS*). The haem-containing enzyme seems to be more common in prokarya members and it has been described from different bacteria such as many *Pseudomonas *and *Paracoccus *species. The enzymes containing Cu and haem never coexist within the same bacterial species [58]. Up to now, only two respiratory nitrite reductases have been characterised from a haloarchaea member; these are the enzymes from *Har marismortui *and *Hfx denitrificans*, which contain two Cu centres, and in both cases, the protein is encoded by the *nirK *gene. It has been suggested that the physiological electron donor for this protein could be halocyanin, a blue Cu-protein present in some Archaea [59]. This issue should be also addressed in *Hfx mediterranei*.

#### NO, N_2_O, N_2 _production

Nitric oxide (NO), the product of the respiratory nitrite reductases, is a toxic compound that is reduced to N_2_O by nitric oxide reductases (Nor) immediately after it has been generated. Different Nor enzymes from fungal denitrifiers and denitrifying bacteria have been analysed. In some cases, Nor is a soluble monomeric enzyme belonging to the cytochrome P-450 family [60]. In other microorganisms, Nor has been described as a membrane complex of a 17 kDa cytochrome *c *and a 38 kDa cytochrome *b *with 12 transmembrane regions. This enzyme is known such as cNor. It is not clear which class of Nor evolved first and it would thus be informative to characterise some Nors from denitrifying Archaea [54]. With regard to halophilic archaea, there is evidence for the presence of Nor (from physiological and biochemical studies) only in *Har marismortui *and *Hfx denitrificans *[24].

The last step of denitrification, the reduction of N_2_O to N_2_, is of great environmental importance because it closes the N-cycle. N_2_O is less toxic than NO or nitrite and the vast majority of microorganisms could manage without converting N_2_O into N_2_. However, most denitrifying bacteria contain nitrous oxide reductases (Nos) encoded by *nosZ *gene which reduced N_2_O into N_2_. These enzymes are soluble periplasmic multicopper homodimers that receive electrons from cythorome *c *or pseudoaurin [7].

In some halophilic archaea, such as *Haloarcula *it was described that the predominant gas species produced by denitrification is N_2_O [61]. However, a few years later, other studies suggested that, nitrite and N_2 _were produced during exponential growth. When *Har marismortui *culture enters stationary phase, dinitrogen production ceases, the concentration of nitrite in the medium rapidly decreases and nitrous oxide is accumulated. Other haloarchaea such as *Hfx denitrificans *are able to reduce N_2_O to N_2_, having a complete denitrification pathway [62].

**Figure 4:**
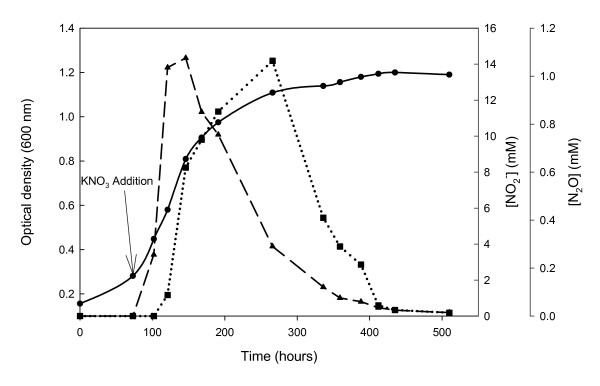
Nitrite and nitrous oxide production by *Hfx mediterranei *cells growing under anaerobic conditions as previously described [15]. (●) Optical density (600 nm) vs. time (hours), (◀) NO_2_^- ^production (mM) vs. time (hours), (■) N_2_O production (mM) vs. time (hours).

The first of this kind of physiological experiment developed with *Hfx mediterranei *suggested that it produces dinitrogen during exponential growth accompanied of the accumulation of low quantities of nitrite and did not produce nitrous oxide [17]. However, recently, the production of N_2_O has been quantified from *Hfx mediterranei *cultures (Fig 4).

### Operon structure of denitrification genes in *Hfx mediterranei*

Denitrification and other anaerobic respiratory processes are alternative metabolic pathways developed by facultative microorganisms to obtain energy for growth under anoxic conditions, and therefore they are usually repressed by oxygen [63]. The control of the genes involved in this pathway is well studied in denitrifying bacteria. Although, the molecular basis of the regulatory networks of denitrification is beginning to emerge in halophilic archaea, they are still not well resolved. Molecules such us O_2_, NO_3_^-^, NO_2_^- ^and NO, act as signals to induce the regulation of this pathway. Related to this, more than one type of regulatory protein is involved in sensing the molecules cited above in bacteria: i) oxygen sensors (FixL and FNR) [64]; ii) nitrate and nitrite sensors (NarXL, NarPQ and NarR) [65]; iii) nitric oxide sensors (NNR and NoR); iv) redox sensors (Reg regulon) [66] and v) NiR and NosR proteins (essential proteins for expression and transcription of the *nos *and *nir *gene cluster) [67,68].

There are only few *in silico *studies which reveal that potential regulators involved in bacterial nitrate reduction (cited above) are not present in the few archaeal genomes sequenced to date. This fact suggests that a novel regulatory system could operate in archaea. Some studies in methanogenic archaea demonstrated that Mo also regulates the expression of the genes required for archaeal molybdoproteins [69].

In general, bacterial and archaeal respiratory nitrate systems also differ in the organization of the genes [70]. The main *narGHJI *operon organization is conserved in most bacteria; nevertheless halophilic archaea *nar *genes do not conserve this organization. In *Har marismortui*, for example, two small ORFs and a NarJ homologue are located downstream from the narGH genes, and putative genes encoding an iron-sulphur protein and a cytochrome *b *are located upstream from narGH [16]. Similar organization has been found in *Hfx mediterranei *Nar system. In this case, the *nar *operon encodes eight open reading frames (ORF1, *narB*, *narC*, ORF4, *narG*, *narH*, ORF7 and *narJ*). ORF1 (Q703I0), ORF4 (Q703H7) and ORF7 (Q703H3) are open reading frames showing similarity with small proteins (molecular mass around 20 kDa) involved in electron transport. *narB *(Q703H9) codes for a 219-amino-acid-residue Rieske iron-sulfur protein. The ORF 1, 4 and 7 and NarB are predicted to form a quinol-dependent electron transfer system that could be coupled to free energy transduction by a Q-cycle mechanism. *narC *(Q703H8) encodes a protein of 486 amino acid residues identified by databases searches as cytochrome-*b *(*narC*). The *narG *(Q703H6) gene encodes a protein with 983 amino acid residues and is identified as a respiratory nitrate reductase catalytic subunit (*narG*). NarH protein has been identified as an electron transfer respiratory nitrate reductase subunit (*narH*, Q703H5)). The last ORF encodes a chaperonin-like protein (*narJ*, Q703H4) of 242 amino acid residues [15].

As the *nar *genes were present before the phylogenetic divergence of bacteria and archaea, it can be assumed that respiratory nitrate reductase played a key role in energy metabolism during pre-oxic times.

We used RT-PCR to determine the effect of anaerobic conditions and nitrate in the expression of the *na*r operon in *Hfx mediterranei*. In summary, regulation of *nar *genes occurs at transcriptional level, and is influenced by oxygen-limiting conditions and the presence of nitrate. No regulatory protein could be identified in the vicinity of the *nar *operon, but the presence of a set of palindromic sequences in the promoter suggests that the transcriptional regulation occurs via protein binding. No known regulatory sequence patterns have been identified, indicating that this transcriptional regulation could be novel in the denitrifier organisms.

### *Hfx mediterranei *role in bioremediation processes

Denitrification is important process in agriculture where it results in the loss of nitrate fertilizers from fields and in waste-treatment processes where nitrate must be removed from waste waters before release into the environment. Nitrate and nitrite have important agricultural, environmental, and public health implications [4]. The manufacturing of chemicals compounds (pesticides, herbicides, explosives, etc.) usually generates effluents containing complex mixtures of salts and nitrate or nitrite. The capacity of microorganisms to degrade organic pollutants is severely limited by their ability to survive or proliferate in these waste waters [71]. The increase of salinity and nitrate/nitrite concentrations in soils and ground waters in the last few decades has focused much attention on the physiological and molecular mechanisms involved in salt-stress tolerance and nitrate metabolism by microorganisms [72]. Microorganisms are in general sensitive to low nitrate and nitrite concentrations. The inhibitory effect of these nitrogen compounds is due to the extreme toxicity of nitrite and nitric oxide produced upon nitrate reduction as it has been mentioned before.

Physiological studies carried out with Hfx mediterranei have revealed that it is resistant to very high nitrate (up to 2 M) and nitrite (up to 50 mM) concentrations (unpublished data). The nitrite concentration present in the cultures employed is one of the highest yet described for a prokaryotic microorganism. The nitrite present in the culture medium was removed during the growth of Hfx mediterranei; in fact, at the final of the stationary phase of growth (OD = 2.2), only 20% of the nitrite present in the culture was still present in media in which the initial concentration was around 50 mM. So it is possible to envisage that this haloarchaeon could be applied in water bioremediation processes with the purpose to repair the damage caused by the excessive use of fertilizers in agricultural activities. This application could be beneficial in regions such as Comunidad Valencia or Murcia (Spain), where the water tables contain high nitrate and nitrite concentrations due to fertilization practices [73].

## Abbreviations

Nir: Assimilatory nitrite reductase; Nas: Assimilatory nitrate reductase; Nar: Respiratory nitrate reductase; NIR: Respiratory nitrite reductase; Nor: Nitric oxide reductase; Nos: Nitrous oxide reductase; GS: Glutamine synthetase; GOGAT: Glutamate synthase; GDH: Glutamate dehydrogenase; SHE: Standard hydrogen electrode.
